# The Proteolipid Protein Promoter Drives Expression outside of the Oligodendrocyte Lineage during Embryonic and Early Postnatal Development

**DOI:** 10.1371/journal.pone.0019772

**Published:** 2011-05-10

**Authors:** John-Paul Michalski, Carrie Anderson, Ariane Beauvais, Yves De Repentigny, Rashmi Kothary

**Affiliations:** 1 Ottawa Hospital Research Institute, Ottawa, Ontario, Canada; 2 Department of Cellular and Molecular Medicine, University of Ottawa, Ottawa, Ontario, Canada; 3 Department of Medicine, University of Ottawa, Ottawa, Ontario, Canada; Virginia Commonwealth University, United States of America

## Abstract

The proteolipid protein (*Plp*) gene promoter is responsible for driving expression of one of the major components of myelin – PLP and its splice variant DM-20. Both products are classically thought to express predominantly in oligodendrocytes. However, accumulating evidence suggests *Plp* expression is more widespread than previously thought. In an attempt to create a mouse model for inducing oligodendrocyte-specific gene deletions, we have generated transgenic mice expressing a Cre recombinase cDNA under control of the mouse *Plp* promoter. We demonstrate *Plp* promoter driven Cre expression is restricted predominantly to mature oligodendrocytes of the central nervous system (CNS) at postnatal day 28. However, crosses into the *Rosa26^LacZ^* and *mT/mG* reporter mouse lines reveal robust and widespread Cre activity in neuronal tissues at E15.5 and E10.5 that is not strictly oligodendrocyte lineage specific. By P28, all CNS tissues examined displayed high levels of reporter gene expression well outside of defined white matter zones. Importantly, our study reinforces the emerging idea that *Plp* promoter activity is not restricted to the myelinating cell lineage, but rather, has widespread activity both during embryonic and early postnatal development in the CNS. Specificity of the promoter to the oligodendrocyte cell lineage, as shown through the use of a tamoxifen inducible *Plp-CreER^t^* line, occurs only at later postnatal stages. Understanding the temporal shift in *Plp* driven expression is of consequence when designing experimental models to study oligodendrocyte biology.

## Introduction

Proteolipid protein (PLP), a tetraspan membrane protein, is the most abundant component of CNS myelin. It is encoded by a highly conserved 17 Kb gene (*Plp*) containing seven exons. By means of an alternate splicing event, a second protein, DM-20, is also generated. *Plp*, though active at low levels during embryonic development, is expressed maximally in mature myelinating cells, while *DM-20* is expressed earlier during embryonic development in both neural, and subsequently, oligodendrocyte progenitors [1], [2], [3]. Together, PLP and DM-20 play an important role in regulating proper oligodendrocyte maturation and myelin compaction/stability over time [4], [5].

The *Plp* promoter and its regulatory elements, located in exon 1 and intron 1 of the gene, were previously shown to dictate expression specifically to oligodendrocytes [6], [7], [8] and Schwann cells [9], [10] at various stages of development. Although the promoter is generally considered oligodendrocyte lineage specific, accumulating evidence suggests a *Plp* expression pattern outside of defined white matter zones; included are both glial and neuronal CNS subpopulations, implying a role for *Plp* in activities independent of myelination [11], [12], [13], [14], [15], [16].

To study *Plp* promoter driven expression patterns across time in CNS tissues, we have generated and characterized transgenic mice expressing full length Cre cDNA under control of the mouse *Plp* promoter (*Plp*-*Cre*). We did not necessarily expect the transgene to completely faithfully mimic the endogenous *Plp* gene expression due to the phenomenon of position-effect in transgenic mice, but rather we wished to test whether such cassettes can provide cell specificity in a temporal fashion. These mice demonstrate a transient spatio-temporal expression pattern for *Plp-Cre*. As expected, at P28 the *Plp* promoter drove Cre expression predominantly in mature oligodendrocytes; however, by crossing the *Plp-Cre* line to either the *Rosa26^LacZ^* [17] or the *mT/mG* [18] reporter mice, a more promiscuous expression pattern was observed during embryonic and early postnatal stages of development. Specificity of the promoter to the oligodendrocyte cell lineage, as shown through the use of a tamoxifen inducible *Plp-CreER^t^* line [19], occurs only at later postnatal stages.

Our work reinforces previous studies supporting a role for the *Plp* promoter in the nervous system outside of myelinating cell lineages, particularly early in development. Consequently, the promoter construct should be used with caution in studies requiring genetic modifications strictly in myelinating cells, especially when embryonic and early postnatal time points are involved.

## Materials and Methods

### Ethics Statement

The mice were cared for according to the Canadian Council on Animal Care (CCAC) guidelines. Ethical approval for experiments conducted was obtained from the University of Ottawa Animal Care Committee under protocol approval OGH-118 and OGH-119.

### Transgenic and Reporter Mice

The *Cre* cDNA (kindly provided by Dr. Robin Parks, Ottawa Hospital Research Institute) was inserted into the *Plp* promoter cassette, consisting of exon 1 and intron 1 of the mouse *Plp* gene [7]. The resulting transgene construct was microinjected into one-cell mouse embryos. Tail biopsies were obtained from potential founder mice, DNA was extracted and transgenic mice were identified by PCR amplification using sense oligo 5′ TGG GTG TTG GTT TTT GGA GA 3′ (specific to the *Plp* promoter) and antisense oligo 5′ CGC ATA ACC AGT GAA ACA GCA 3′ (specific to the *Cre* cDNA). Positive founder mice were bred with C57BL/6 mice (obtained from Charles River) to establish two independent transgenic lines. *Plp-CreER^t^* mice were kindly provided by Dr. Brian Popko [19]. One mg of tamoxifen (20 µL of a 50 mg/ml solution) was administered intraperitoneal once a day for 5 consecutive days to P16 mice. A single 1 mg injection was given to P4 mice. Cre protein distribution was assessed by crossing transgenic mice into the *Rosa26^LacZ^* reporter line [17] or to a floxed stop tdTomato-EGFP (*mT/mG*) fluorescent reporter mouse line [18]. Animals homozygous for the *Rosa26^LacZ^* or *mT/mG* locus were crossed to mice heterozygous for the *Cre* transgene, generating a final line of mice heterozygous for both the *Cre* and respective reporter transgenes. The genotype of offspring mice was confirmed by PCR.

### RT-PCR Analysis

For reverse transcription-polymerase chain reaction (RT-PCR) analysis, RNA was isolated from transgenic P4 and P28 tissues from the two *Plp-Cre* founder lines. To produce cDNA, equal amounts of RNA were reverse-transcribed in a standard reaction with MuLV reverse transcriptase (Invitrogen). PCR amplification using the sense oligo 5′ CCT TCC AGC TGA GCA AAG TC 3′ (specific to *Plp* exon 1), and the antisense oligo 5′ CGC ATA ACC AGT GAA ACA GCA 3′ (specific to the *Cre* cDNA) yielded a 440 nucleotide fragment. Primers were chosen to flank the intronic region of the construct to selectively amplify the RNA transcript and prevent amplifying any contaminating genomic DNA. The reaction began with a 3 min incubation time at 94°C followed by 30 cycles of 45 sec at 94°C, 45 sec at 55°C, 1 min at 68°C, with a final extension time of 10 min at 68°C. Amplification of actin cDNA served as a control. The PCR products were electrophoresed on a 1.5% agarose gel containing ethidium bromide, and amplified fragments were visualized under UV transillumination.

### Immunohistochemistry

Mice were anesthetized with avertin and perfused transcardially with 4% paraformaldehyde (PFA). Brains, spinal cords and optic nerves were dissected in PBS, fixed with 4% PFA overnight at 4°C, cryoprotected in 30% sucrose overnight at 4°C, then frozen in a 1∶1 mixture of 30% sucrose:OCT (Sakura, CA). Cryostat sections of 10 µm thickness were obtained and stored at −20°C until use. Sections were postfixed in 70% ethanol for 5 min, rinsed with PBS for 10 min, and incubated in warm citrate buffer (10 mM citric acid, 26 mM NaOH, pH 6) for 10 min. Sections were then blocked using TBLS (0.5 mM Tris-HCl pH 7.4, 0.0085% NaCl, 0.01% BSA, 0.009% L-lysine, and 10% sodium azide) with 20% goat serum and 0.3% Triton-X-100 for 1 hour. Primary antibodies for CC-1 (1∶10, Abcam, MA), GFAP (1∶500, chemicon), Ng2 (1∶250, Millipore), NeuN (1∶100, Chemicon), and Cre (1∶100, Novagen) were diluted in TBLS and incubation was for 1 hour at RT and overnight at 4°C, respectively. Secondary antibodies were used at a dilution of 1∶200 (Invitrogen), and Hoechst counterstain at 1∶10,000. Slides were mounted with a fluorescence mounting medium (Dako North America, Inc.) and sections analyzed by fluorescence microscopy using a Zeiss Axioplan microscope or Zeiss Confocal microscope (LSM 510 Meta DuoScan). Cells were considered to be Cre positive only if they had a clear nuclear staining pattern co localizing with DAPI. All images for co labeling analysis were done using the Zeiss Confocal microscope set at a 1 µm slice capture.

### β-galactosidase staining

Heterozygous male *Plp-Cre* or *Plp-CreER^t^* mice were bred with female *Rosa26^LacZ^* homozygotes. The Cre positive offspring should harbor cells expressing a functional Cre-recombinase enzyme; hence allowing expression of *lacZ* driven by the ROSA26 promoter. Cre-positive *Rosa26^LacZ^* heterozygous progeny were analyzed for β-galactosidase enzyme activity, with Cre-negative *Rosa26^LacZ^* heterozygotes serving as controls. Mice were anesthetized with avertin and perfused transcardially using 0.2% glutaraldehyde with 1.25 mM EGTA pH 7.3 and 2 mM MgCl_2_ in PBS. Tissues (brain, spinal cord, sciatic nerve, optic nerve) or embryos were then postfixed in 0.2% glutaraldehyde solution for 1 to 4 additional hours at 4°C, then rinsed 3 times with PBS. Tissues were incubated from 2 hours to overnight at 37°C in X-Gal staining solution consisting of 5 mM potassium ferricyanide, 5 mM potassium ferrocyanide, 2 mM MgCl_2_, 0.02% Nonidet P-40, and 1 mg/ml of 5-bromo-4-chloro-3-indolyl-β-D-galactopyranoside (X-Gal) in 0.1 M phosphate buffer, pH 8. Samples were then washed 3 times with PBS and whole mount photographs were taken.

## Results

### Generation of Plp-Cre transgenic mice

**Figure 1:**
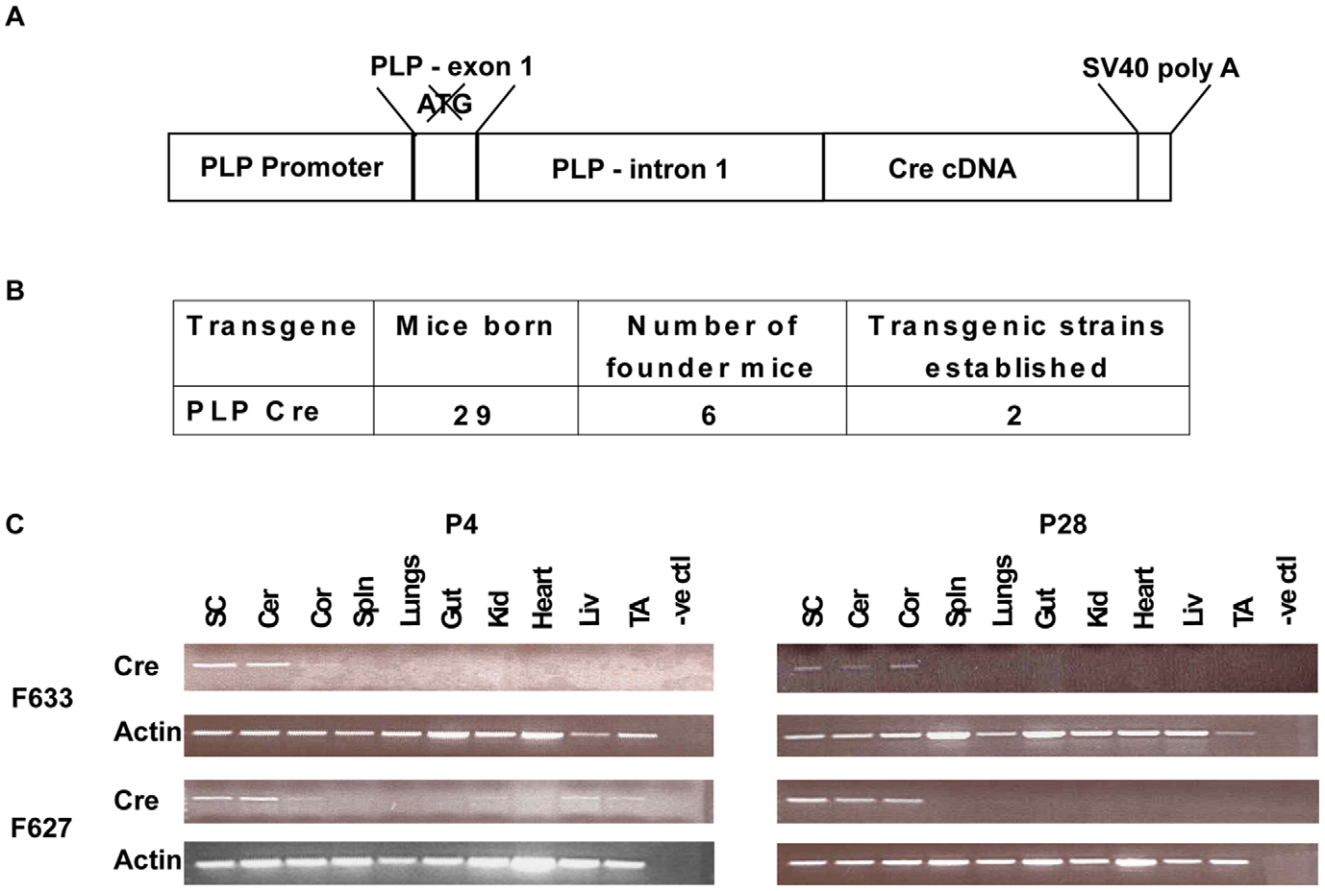
Schematic and expression profile of *Plp-Cre* transgene. **A.** Schematic representation of the *Plp-Cre* transgene construct. The mouse *Plp* promoter is used to drive expression of the *Cre* cDNA. The initiating ATG within exon 1 has been mutated to prevent initiation of translation within the promoter region. **B.** Summary of *Plp-Cre* transgenic mice generated. **C.** RT-PCR analysis of the tissue expression profile of the *Plp-Cre* transgene for both founder lines. At P28, both lines adhered to a strictly CNS-specific expression pattern, with transcripts detected in the spinal cord, cerebellum and cortex. At P4, the transgene transcript in line F633 was detected solely in CNS tissues, whereas line F627 displayed a more widespread expression pattern, with transcripts also detected in kidney, liver and TA muscle. SC  =  spinal cord, Cer  =  cerebellum, Cor  =  cortex, Spln  =  spleen, Kid  =  kidney, Liv  =  liver, TA  =  tibialis anterior muscle. Actin mRNA amplification was used as control.

To direct oligodendrocyte specific expression, full-length *Cre* cDNA was placed under control of the mouse *Plp* promoter (Fig. 1A). The promoter region consists of exon 1 and intron 1 of the *Plp* gene, shown previously to be sufficient for mimicking the endogenous *Plp* activity (7). Six of 29 potential founders tested positive for the transgene construct with two successfully breeding and transmitting the transgene to subsequent generations (Fig. 1B). From these two founders, independent lines 627 and 633, described herein, were generated.

### The Plp-Cre transgenic mice display CNS-specific expression

Specificity of *Plp-Cre* expression to CNS tissues in lines 627 and 633 was initially assessed by RT-PCR. To compare early and later stage transgene expression, total RNA was isolated from selected tissues at P4 and P28. At P28, both lines exhibited a CNS-specific expression pattern, with transcripts detected in, for example, spinal cord, cerebellum and cortex (Fig. 1C). At P4, transgene expression was also detected in CNS tissues (Fig. 1C). However, at this stage, mice from line 627 also expressed the Cre transgene in kidney, liver, and skeletal muscle (Fig. 1C). Nevertheless, it should be noted that for the purpose of this study the transgene was consistently expressed at higher levels in CNS tissues relative to all other tissues examined.

### Plp-Cre transgene expression displays selectivity to oligodendrocytes in later postnatal stage mice

**Figure 2:**
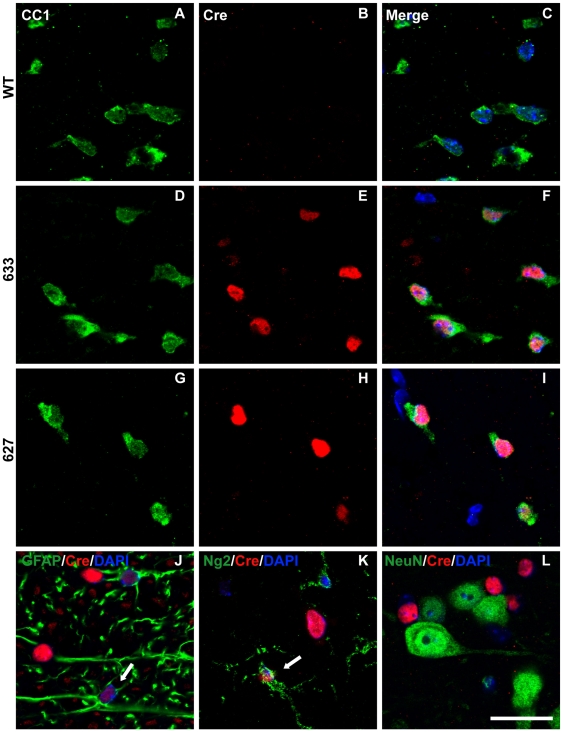
Cre-recombinase is predominantly expressed in oligodendrocytes of P28 *Plp-Cre* mouse spinal cord. **A**–**I.** Ventral white matter regions were double-stained with antibodies specific to CC-1 as a marker for mature oligodendrocytes (green), Cre (red) and counterstained with DAPI. Cre was not detected in WT spinal cord sections (B). Sections from transgenic mice of both F633 (D–F) and F627 (G–I) lines exhibited a similar pattern of Cre expression. High levels of Cre recombinase protein was detected in the CC1-positive oligodendrocytes. **J.** Ventral white matter region stained for GFAP (green) as a marker for astrocytes, Cre (red) and counterstained with DAPI. A small number of Cre-positive cells co-labeled with GFAP (arrow). **K.** Ventral white matter region stained for Ng2 (green) as a marker for OPCs, Cre (red) and counterstained with DAPI. A small number of Cre-positive cells co-labeled with Ng2 (arrow). **L.** Ventral grey matter region stained for NeuN (green) as a neuronal marker, Cre (red) and counterstained with DAPI. There were no NeuN positive cells that co-labeled with Cre. Scale bar  = 20 µm.

**Figure 3:**
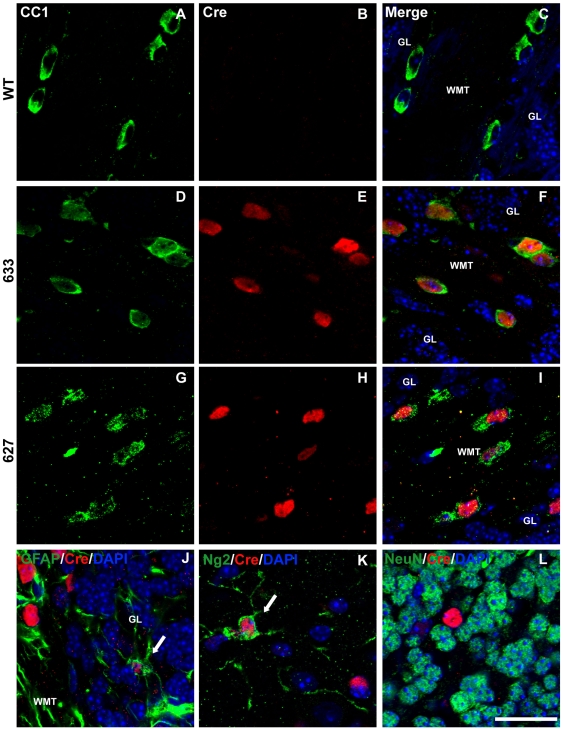
Cre-recombinase is predominantly expressed in oligodendrocytes of P28 *Plp-Cre* mouse cerebellum. **A**–**I.** Cerebellar sections were double-stained with antibodies specific to CC-1 as a marker for mature oligodendrocytes (green), Cre (red) and counterstained with DAPI. Cre was not detected in WT cerebellar sections (B). Sections from transgenic mice of both F633 (D–F) and F627 (G–H) lines exhibited a similar pattern of Cre expression. High levels of Cre recombinase protein was detected in the CC1-positive oligodendrocytes. **J.** Cerebellar section stained for GFAP (green) as a marker for astrocytes, Cre (red) and counterstained with DAPI. A small number of weakly stained Cre-positive cells co-labeled with GFAP (arrow). **K.** Representative example within the molecular layer of cerebellar section stained for Ng2 (green) as a marker for OPCs, Cre (red) and counterstained with DAPI. A small number of weakly stained Cre-positive cells co-labeled with Ng2 (arrow). **L.** Representative example within granular layer of cerebellar section stained for NeuN (green) as a neuronal marker, Cre (red) and counterstained with DAPI. There were no NeuN positive cells co-labeled with Cre. GL  =  granular layer, WMT  =  white matter tract. Scale bar  = 20 µm.

Immunocytochemistry was performed to assess specificity of Cre protein expression to oligodendrocytes. Cerebellar, spinal cord, and optic nerve sections from P4 and P28 *Plp-Cre* transgenic mice and their wild type littermates were examined. An antibody against CC-1 was used as a marker for mature oligodendrocytes. In both *Plp-Cre* lines (627 and 633), all neural tissues examined at P28 expressed the Cre protein. The majority of Cre-positive cells also labeled positive for CC-1 (see Table 1), indicating specificity of the *Plp* promoter to mature oligodendrocytes at this stage in postnatal development (spinal cord Fig. 2D–I, cerebellum Fig. 3D–I, and optic nerve Fig. S1D–I). As expected, we did not detect any Cre-positive cells in tissues from WT littermates (Fig. 2A–C, Fig. 3A-C, and Fig. S1A–C). There were a few faintly labeled Cre-positive cells that also co-labeled with the astrocytic marker GFAP (Fig. 2J, Fig. 3J and Fig. S1J) as well as with Ng2, a marker for oligodendrocyte progenitor cells (OPCs) (Fig. 2K, Fig. 3K and Fig. S1K) (summarized in Table 1). In comparison, there were no Cre-positive cells that co-labeled with NeuN, a marker for mature neurons, in the tissues examined (Figs. 2L and 3L).

**Table 1:** Distribution of Cre-positive cell types in P28 *Plp-Cre* CNS tissues.

	% of CC-1+cells that are Cre+^a^	% of Cre+cells that are CC-1+^a^	% of Cre+cells that are Ng2+	% of Cre+cells that are GFAP+^a^	% of Cre+cells that are NeuN+
Spinal Cord	88.9%±5.4	95.8%±6.8	10.8%±0.7	10.6%±2.2	0
Cerebellum	86.0%±4.8	90.0%±9.2	5.8%±4.3	8.6%±5.8	0
Optic Nerve	91.2%±6.4	88.7%±2.8	3.8%±2.5	3.3%±0.2	n.d.

a There are most likely Cre-positive cells which stain for GFAP and CC-1. These cells are assumed to be GFAP astrocytes, and not oligodendrocytes.

n.d. = not determined

**Figure 4:**
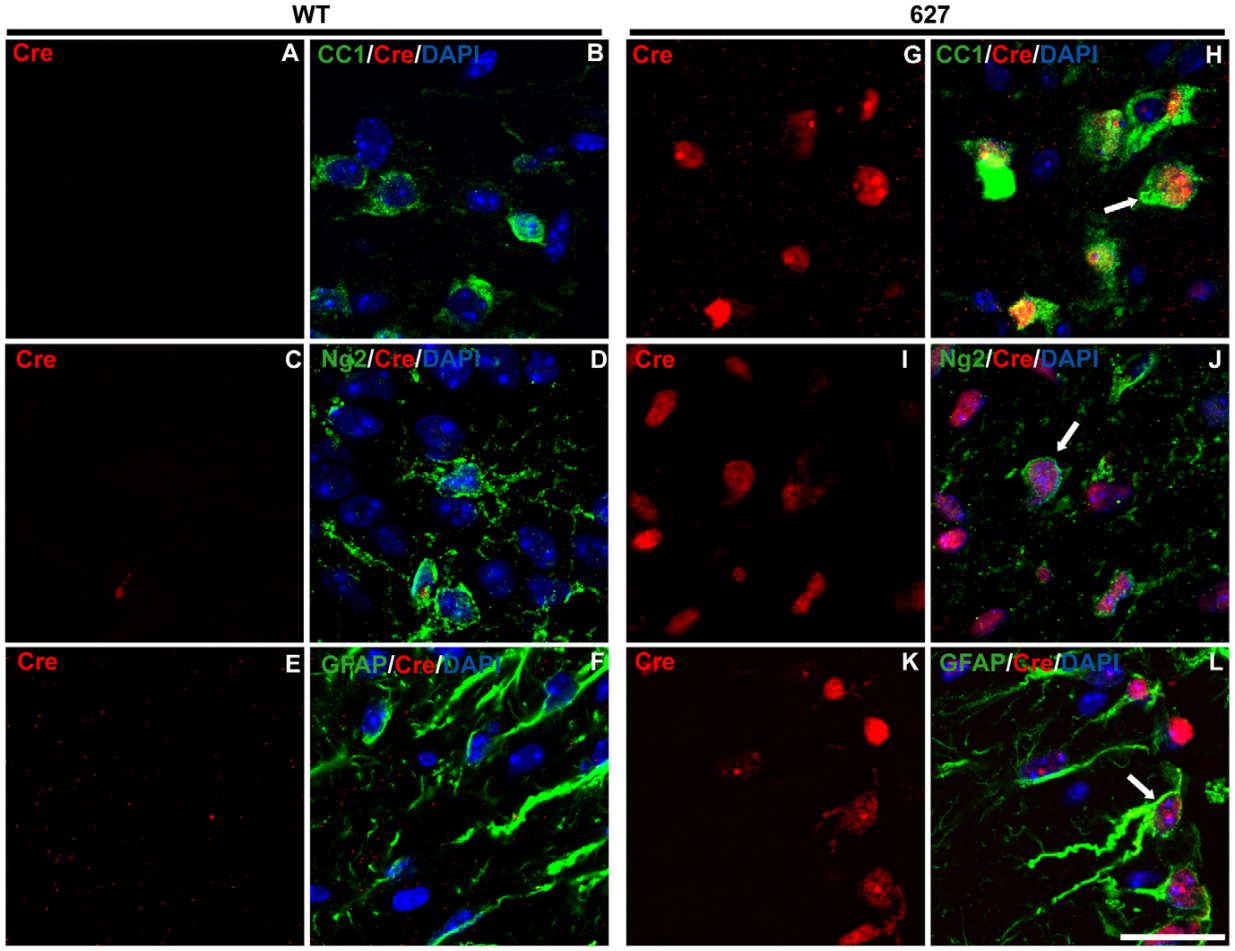
Cre-recombinase is expressed both in and outside the oligodendrocyte lineage in spinal cords of P4 *Plp-Cre* mice. **A**–**L.** Ventral spinal cord sections from WT and *Plp-Cre* line 627 mice were double-stained with antibodies specific to Cre (red) and either CC-1 (green) as a marker for mature oligodendrocytes (A–B, G–H), Ng2 (green) as a marker for OPCs (C–D, I–J), or GFAP (green) as a marker for astrocytes (E–F, K–L), and counterstained with DAPI. Cre was not detected in WT sections (A–F). As expected, many Cre-positive cells were CC-1-positive, however, a large percentage also co-stained for Ng2 and GFAP. Examples of Cre and glial marker co-labeling are denoted by arrows. WM  =  white matter, GM  =  grey matter. Scale bar  =  20 µm.

**Figure 5:**
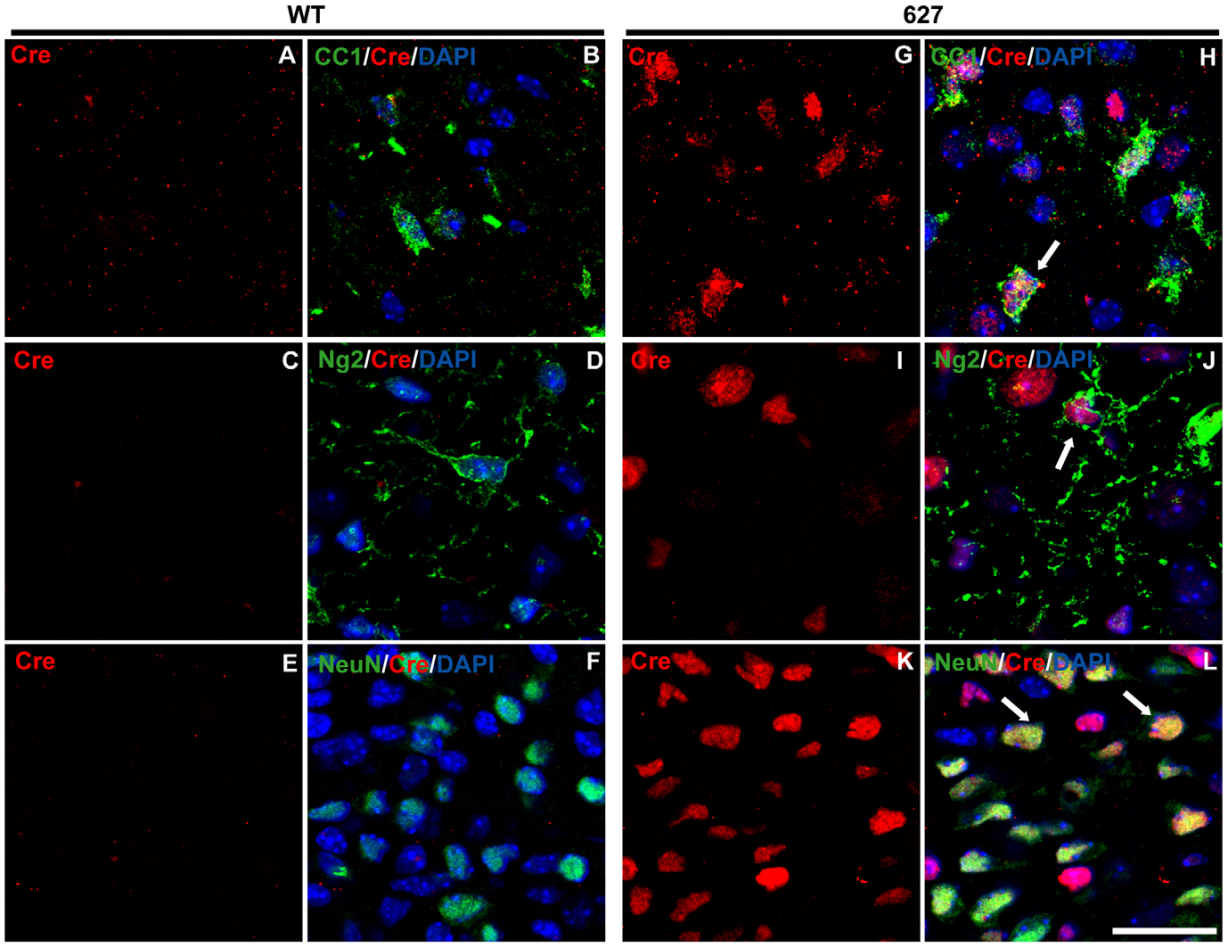
Cre-recombinase is expressed both in and outside the oligodendrocyte lineage in the cerebellum of P4 *Plp-Cre* mice. **A–D**, **G–J**. Deep cerebellar white matter regions from WT and *Plp-Cre* line 627 mice were double-stained with antibodies specific to Cre (red) and either CC-1 (green) as a marker for mature oligodendrocytes (A–B, G–H), or Ng2 (green) as a marker for OPCs (C–D, I–J) and counterstained with DAPI. Cre was not detected in WT sections (A–D). A large percentage of Cre-positive cells within the region co-stained for CC-1 and Ng2. **E–F**, **K–L**. Cerebellar sections of the developing granular layer from WT and *Plp-Cre* line 627 mice were double-stained with antibodies specific to Cre (red), the neuronal marker NeuN (green) and counterstained with DAPI. Cre was not detected in WT sections (E–F). Many Cre-positive cells co-stained for NeuN. Examples of Cre-positive cells co-labeling with neuronal or glial markers are denoted by arrows. Scale bar = 20 µm.

At P4, Cre protein was detected in both spinal cord (Fig. 4, Fig. S2, Fig. S3) and cerebellum (Fig. 5, Fig. S4, Fig. S5) of *Plp-Cre* transgenic mice. However, unlike the situation at the P28 stage, Cre expression was often more widespread, with many Cre-positive cells outside of the oligodendrocyte lineage, especially in the cerebellum (see Table 2).

**Table 2:** Distribution of Cre-positive cell types in P4 *Plp-Cre* CNS tissues.

		% of CC-1+cells that are Cre+^a^		% of Cre+cells that are CC-1+^a^	% of Cre+cells that are Ng2+		% of Cre+cells that are GFAP+^a^			% of Cre+cells that are NeuN+
Spinal Cord	55.0%±11.2		92.0%±9.1			34.4%±3.6		33.0%±7.4	0	
Cerebellum	80.6%±6.8^b^		46.7%±10.2^b^			21.4%±6.6^b^		8.4%±5.1^b^	53.3±14.4^c^	

a There are most likely Cre-positive cells which stain for GFAP and CC-1. These cells are assumed to be GFAP astrocytes, and not oligodendrocytes. This is especially true for P4 spinal cord.

b Cells counted from deep central white matter region of cerebellum.

c Cells counted in developing region of cerebellar granular, Purkinje and molecular cell layers.

In the spinal cord, the most intensely stained Cre-positive cells localized predominantly to emerging white matter tracts, and co-labeled with the oligodendrocyte marker CC-1 (Fig. 4A–B, G–H, Fig. S2A–C). Approximately a third of Cre-positive cells co-labeled with Ng2 (Fig. 4C–D, I–J, Fig. S2D–F). However, a considerable number of Cre-positive cells also were positive for the astrocyte marker GFAP, both in white and grey matter regions (Fig. 4E–F, K–L, Fig. S2G–I). As the cumulative percentage of Cre-positive cells expressing markers for CC-1 and GFAP exceeds 100%, it is suggested that GFAP positive cells are often also labeled by CC-1 at P4 (these cells would be considered astrocytes) (see Table 2). There was no co-labeling of Cre-positive cells with the neuronal marker NeuN at this time point (Fig. S3).

In the P4 cerebellum, CC-1 positive cells were localized to the deep central white matter region, the zone of initial myelination. Approximately half (see Table 2) of the central white matter regions Cre-positive cells were CC-1 positive (Fig. 5A–B, G–H, Fig. S4A–C). Cre-positive cells within the region also co-labeled with Ng2 (Fig. 5C–D, I–J, Fig. S4D–F), and a small number with GFAP (Fig. S5). Most striking was the Cre staining pattern in the developing granular, Purkinje and molecular cell layers. Almost no CC-1 positive cells were identified and less than 10% of Cre-positive cells within this region co-stained for Ng2 in both transgenic lines (data not shown). Approximately half (see Table 2) of all Cre-positive cells co-labeled with NeuN (Fig. 5E–F, K–L, Fig. S4G–I), suggesting a large population of neurons within the granular, Purkinje and molecular cell layers in which the *PLP* promoter is active at P4.

### Inducible model demonstrates PLP promoter expression outside of oligodendrocytes during early postnatal development

The above results suggest a temporal shift in the promoter's expression pattern. To further validate the observed difference in *Plp* promoter activity between early (P4) and late (P28) postnatal time points, we took advantage of an existing line in which *Plp* drives expression of a Cre protein fused to a mutated estrogen receptor (*Plp-CreER^t^*) [19]. This fusion necessitates activation of Cre by tamoxifen. The *Plp* promoter region, similar to our *Plp-Cre* transgenic mice, consists of exon 1 and intron 1 of the endogenous *Plp* gene, and as such, would be expected to drive Cre expression in an identical manner. The *Plp-CreER^t^* mice were crossed to a ROSA26 reporter line (*Rosa26^LacZ^*). *Rosa26^LacZ^* mice express *lacZ* driven by the constitutively active ROSA26 locus promoter [17]. A floxed stop region is located upstream of the *lacZ* coding sequence, therefore necessitating Cre-mediated excision of the stop region to allow for *lacZ* expression. Tamoxifen is thus required to induce translocation of CreER^t^ from the cytoplasm into the nucleus thereby activating *lacZ* expression. For the *Plp-CreER^t^* transgenic line, injections of tamoxifen were given at P16 and P4. The time points were chosen to reflect different stages of oligodendrocyte maturation and the observed temporal shift in *Plp* promoter patterning observed in our *Plp-Cre* mice; that of increasing specificity to the oligodendrocyte lineage as the animal matures from P4 to P28. Injections at P4 would allow for early postnatal *Plp* expression profiling, while the later P16 time point would reflect the increasing oligodendrocyte selectivity of the *Plp* promoter. P16 and P4 tamoxifen injected mice were analyzed at P60 and P28 respectively. Therefore, the reporter-positive cells observed at P60 and P28 are the progenies of *Plp* promoter active cells at P16 and P4. The reporter-positive cells at the stages examined do not necessarily have *Plp* promoter activity.

**Figure 6:**
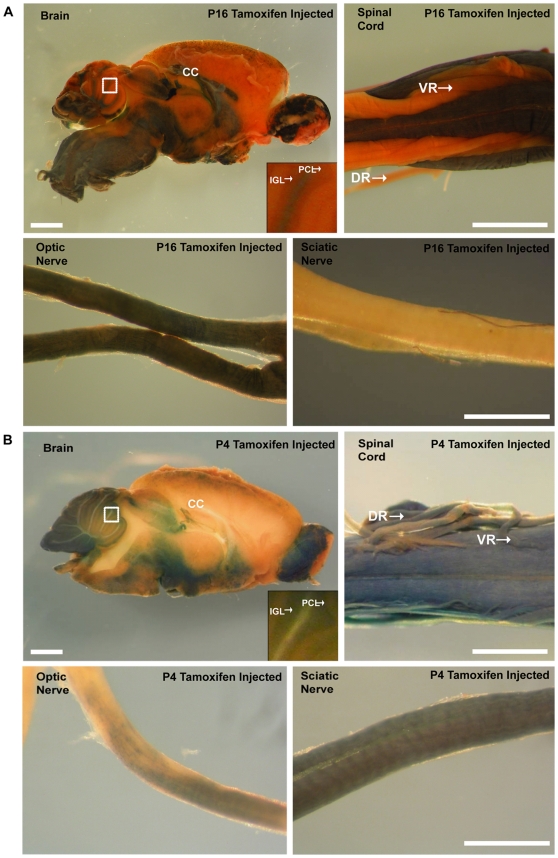
β-galactosidase expression in *Plp-CreER^t^;ROSA26^LacZ^* mice given tamoxifen at either at P16 or P4. **A.** β-galactosidase expression in *Plp-CreER^t^;ROSA26^LacZ^* mice given tamoxifen at P16 and examined by whole mount X-gal staining at P60. In the brain, β-galactosidase activity is predominantly observed in white matter tracts. In the spinal cord, β-galactosidase activity is detected in the dorsal and ventral white matter tracts, but is absent from the ventral and dorsal roots. Finally, β-galactosidase activity is apparent throughout the length of the optic nerve, but not in the sciatic nerve. **B.** β-galactosidase expression in *Plp-CreER^t^;ROSA26^LacZ^* mice given tamoxifen at P4 and examined by whole mount X-gal staining at P28. In the brain, β-galactosidase activity is detectable outside of large white matter tracts. In the spinal cord, β-galactosidase activity is detectable in the dorsal and ventral white matter tracts, and is also present in the ventral and dorsal roots. Finally, β-galactosidase is expressed throughout the length of the optic nerve, albeit at a reduced level, and the sciatic nerve is now positive for β-galactosidase activity. CC  =  corpus callosum, PCL  =  Purkinje cell layer, IGL  =  internal granular layer, VR  =  ventral root, DR  =  dorsal root. Scale bars = 1 mm in A and B Brain, 500 µm in B Spinal Cord.

The pattern of *lacZ* expression, as determined by β-galactosidase activity, in *Plp-CreER^t^;Rosa26^LacZ^* mice differed depending on the time course of tamoxifen administration. In mice given the drug at P16, β-galactosidase expression was similar to that previously described [19], localizing mainly to white matter tracts of the CNS (e.g. in the brain, spinal cord and optic nerve), but not in the PNS (e.g. sciatic nerve) (Fig. 6A). In contrast, when tamoxifen was given to P4 *Plp-CreER^t^;Rosa26^LacZ^* mice, β-galactosidase was expressed both in CNS and PNS tissues (Fig. 6B). Also, β-galactosidase expression in P4 brains was not exclusive to CNS white matter tracts; in the cerebellum β-galactosidase was expressed in the Purkinje cell layer (PCL) and the internal granular layer (IGL) and was not observed in the corpus callosum. The β-galactosidase expression profile observed in the PCL and IGL of P4 injected mice corroborates nicely with our immunocytochemistry for Cre protein performed at the same time point (see Fig. 5E–F, K–L). In both P16 and P4 tamoxifen injected mice, β-galactosidase was expressed in the olfactory bulb, most likely in olfactory ensheathing cells as reported previously [20].

These experiments suggest *Plp* promoter driven expression occurs outside of the oligodendrocyte cell lineage early in postnatal development, and demonstrates increased oligodendrocyte specificity as a result of cellular maturation.

### Plp-Cre transgene expression during embryonic and early postnatal development is not restricted to the myelinating cell lineage

To further assess both the temporal and spatial expression of Cre from early embryonic stages, we crossed our *Plp*-*Cre* transgenic mice to the *Rosa26^LacZ^* line. Brain, spinal cord, and optic nerve tissues were examined from two early developmental time points (E10.5, E15.5). Cells marked by activation of the *lacZ* reporter gene would therefore suggest *Plp* promoter driven Cre expression either before or at the time of analysis.

**Figure 7:**
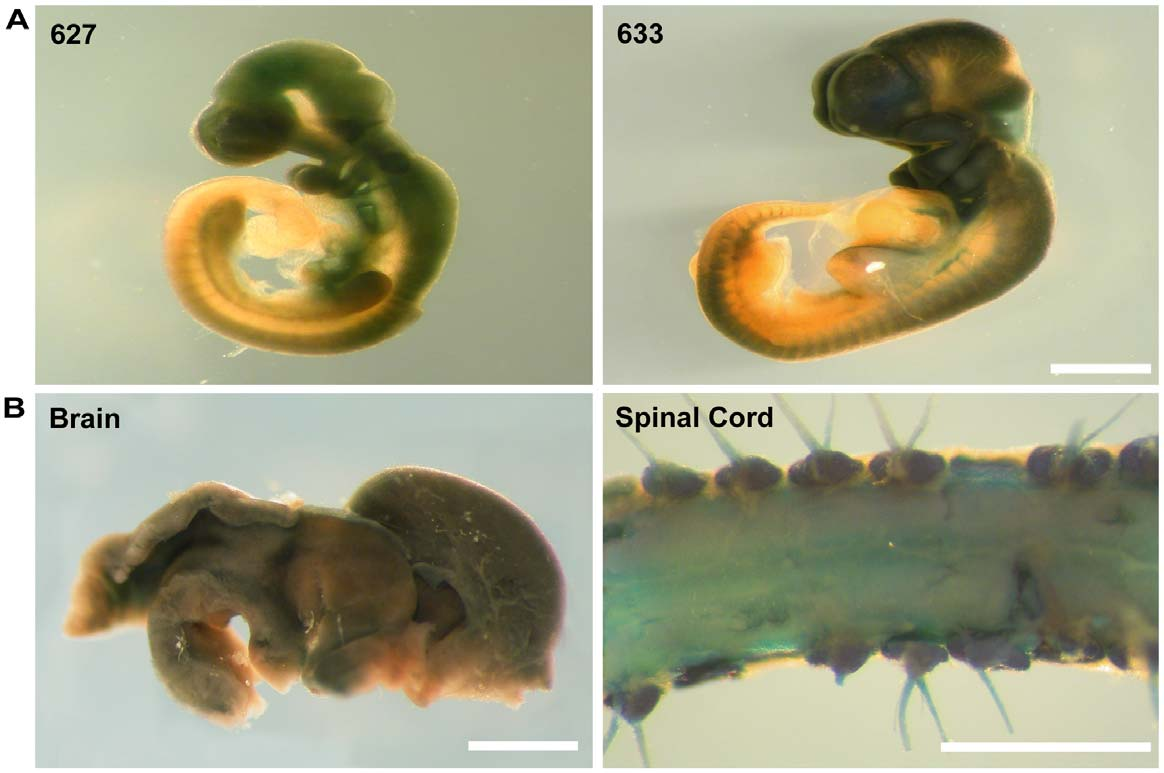
β-galactosidase expression outside of the oligodendrocyte lineage in CNS tissues of *Plp-Cre;ROSA26^LacZ^* mice at E10.5 and E15.5. A. β-galactosidase expression in *Plp-Cre;ROSA26^LacZ^* embryos at E10.5. Shown are representative examples of E10.5 embryos from lines F627 and F633 after whole mount X-gal staining. Both lines show β-galactosidase expression throughout the developing brain and spinal cord. **B**. β-galactosidase expression in *Plp-Cre;ROSA26^LacZ^* mice at E15.5. Shown is an example of whole mount staining of tissues from line F633. β-galactosidase activity was detected throughout both the brain, and ventral and dorsal spinal cord. Scale bars = 2 mm in A and B Brain and Spinal Cord, 1 mm in A and B Optic nerve and Sciatic nerve.

β-galactosidase activity was detected in E10.5 whole mount embryos (Fig. 7A). Robust staining was apparent throughout the telencephalon, optic stalk, mesencephalon, rhombencephalon, and spinal cord as well as in PNS derivatives of the neural crest (Fig. 7A). As the progeny of these cells faithfully inherit the activated *lacZ* gene, it was not surprising to observe widespread β-galactosidase activity in the brains and spinal cords of E15.5 (Fig. 7B) mice. No β-galactosidase expression was observed in either *Plp-Cre* or *Rosa26^LacZ^* mice alone (data not shown).

**Figure 8:**
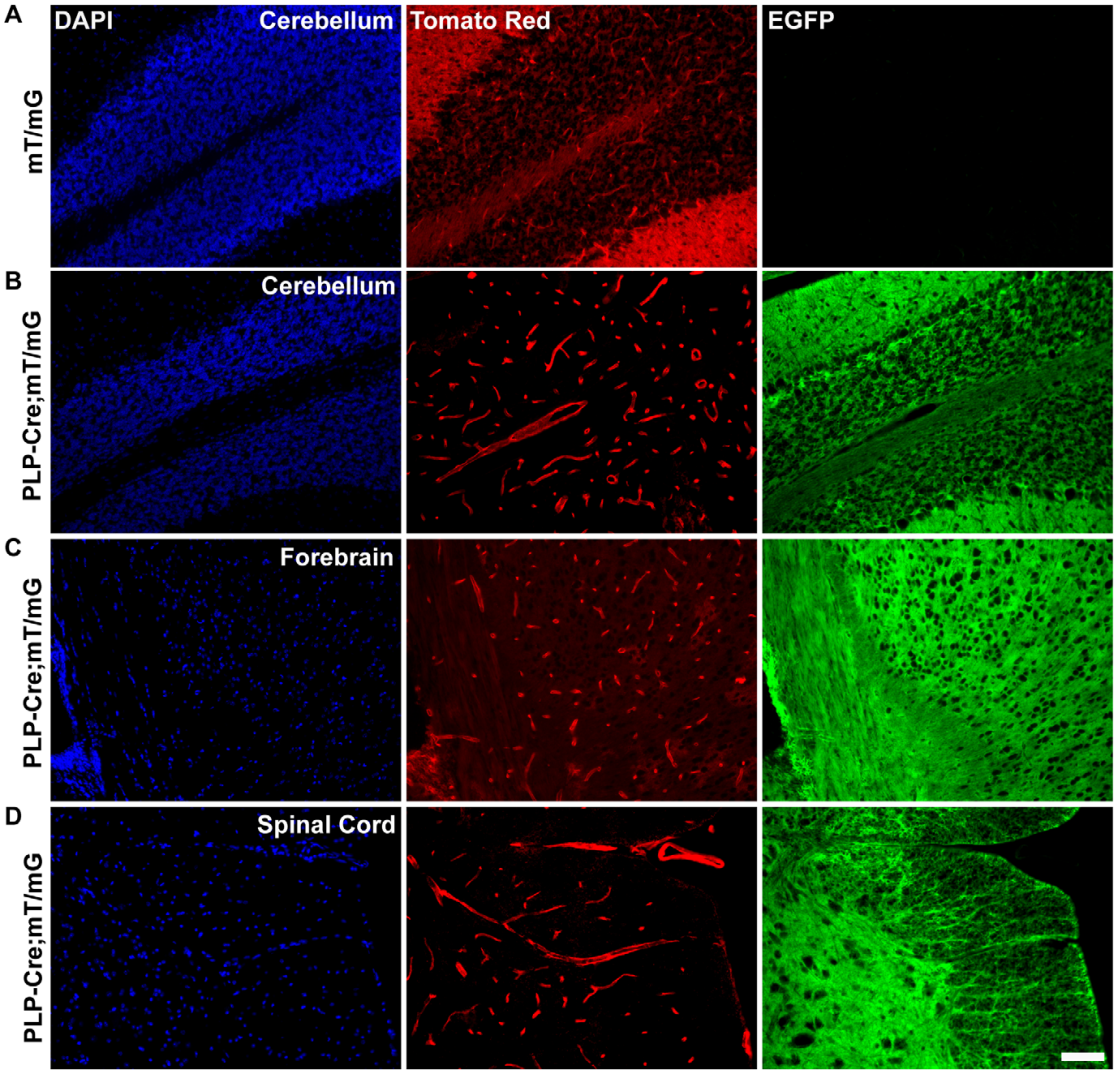
EGFP expression occurs outside of the oligodendrocyte lineage in CNS tissue of P28 *Plp-Cre;mT/mG* mice. **A.** Representative example of *mT/mG* reporter expression in the absence of Cre recombinase in sections of the cerebellum from non-transgenic mice. Only tomato red expression is detected and EGFP expression is absent. **B.** EGFP expression in cerebellum of *Plp-Cre;mT/mG* mice. EGFP is expressed throughout the cerebellum, including the granular, Purkinje and molecular layers. Non-recombined cells, marked by tomato red expression, are limited to the vasculature. **C.** EGFP expression in forebrain region of *Plp-Cre;mT/mG* mice. EGFP is detected throughout the region. Non-recombined cells, marked by tomato red expression, are limited predominantly to vasculature. **D.** EGFP expression in spinal cord of *Plp-Cre;mT/mG* mice. EGFP is detected throughout the spinal cord including gray and white matter zones. Non-recombined cells, marked by tomato red expression, are limited to vasculature. Scale bar = 50 µm.

To allow for higher resolution analysis of *Plp* promoter activity in CNS tissues at a later time point, *Plp-Cre* transgenic mice were crossed to a reporter line expressing *tomato red* and *EGFP* driven by the constitutively active ROSA26 locus (*mT/mG*). A floxed stop region located upstream of the *EGFP* coding sequence necessitates Cre-mediated excision to allow for *EGFP* expression. All cells in which excision of the stop region does not occur would express *tomato red* only. Brain and spinal cord sections from P28 *Plp-Cre;mT/mG* transgenic mice were analyzed. Similar to the *Plp-Cre;Rosa26^LacZ^* double transgenic line, we observed high levels of EGFP expression throughout CNS tissues occurring outside of oligodendrocyte-rich regions (Fig. 8B, C, D). Only very discreet zones remained tomato red positive, localizing predominantly to macro- and micro-vasculature in the cerebellum (Fig. 8B), forebrain (Fig. 8C), and spinal cord (Fig. 8D) areas. A typical example of fluorescent patterning in the absence of Cre is shown as a control (Fig. 8A).

Both the *Rosa26^LacZ^* and *mT/mG* reporter lines clearly demonstrate *Plp* promoter driven Cre activity outside of the oligodendrocyte lineage, beginning as early as E10.5. By P28, with the exception of vasculature, almost all cell types within CNS tissues examined, either during embryonic and/or early postnatal development, have activated the *Plp* promoter region as denoted by high β-galactosidase or EGFP expression levels.

## Discussion

Here we have characterized two independent transgenic mouse lines in which Cre recombinase is expressed under the control of the mouse *Plp* promoter. We demonstrate the *Plp* promoter cassette's capability to drive expression of a Cre recombinase transgene within the CNS, but in a more widespread pattern than initially anticipated. This was particularly true at early developmental stages when the transgene was expressed in neural tissues, and not restricted to the oligodendrocyte lineage, results corroborated by the work of others [3], [14], [16], [21]. During this time, transgene expression was also localized to the PNS (sciatic nerve, ventral and dorsal roots), most likely in Schwann cells. It was only at late postnatal stages that the *Plp-Cre* transgene demonstrated a high level of oligodendrocyte specificity.

At the mRNA level, Cre expression was highly restricted to CNS tissues (within those examined) at early and late stages of postnatal development. At both P4 and P28, transgene expression was detected in cortex, cerebellum, and spinal cord. In addition, at P4, Cre transcript was also detected in the kidney, liver, skeletal muscle, and heart of mice from line 627, albeit at a level lower than that in neural tissues.

At the protein level in the optic nerve, spinal cord, and cerebellum of P28 transgenic mice Cre recombinase was predominantly in mature oligodendrocytes as identified by CC-1 staining. However, at P4, Cre protein expression was more widespread, localizing to granular (Fig. 5E–F, K–L) and Purkinje cell layers within the cerebellum, and throughout spinal cord astrocytes. Previous studies have also identified *Plp* gene products in the external granular and Purkinje cell layers of the developing mouse cerebellum, as well as in grey matter astrocytes of the developing spinal cord [15], [22].

The fact that the *Plp-CreER^t^* transgenic mice have an inducible Cre protein allowed for tracking of the *Plp* promoter activity from specific developmental stages onwards. Indeed, the administration of tamoxifen at specific time points allowed us to follow the fate of cells that were *Plp* positive at the time of administration. The reporter-positive cells observed at later stages are the progeny of *Plp* promoter active cells at the time of tamoxifen administration. Mice given tamoxifen at P4 displayed the *Plp* promoter's promiscuity at this early time point, with the result that β-galactosidase reporter expression was detected in neuronal non-white matter regions at later time points. In stark contrast, the β-galactosidase expression profile in mice receiving tamoxifen at P16 was limited strictly to white matter zones at later stages of analysis. From these studies, a clear *Plp* promoter driven spatio-temporal patterning emerges for the CNS – that of neuronal inclusion early in postnatal development, with later stages defined by increased oligodendrocyte specificity.

Our transgene product, by analysis of *Plp-Cre;Rosa26^LacZ^* mice, proved to be functional in a large range of cell types during embryogenesis. This is dramatically demonstrated in the crosses to the *mT/mG* reporter mice, in which, by P28 all but discreet zones of CNS vasculature expressed excision activated EGFP.

To better understand where and when the *Plp* promoter is active in the CNS, it is important to consider the products of the *Plp* gene. The promoter drives production of both PLP and its splice variant DM-20. The splice site is located within exon three of the gene, producing a transcript product lacking exon 3B [23]. The protein structure of the smaller isoform DM-20 is identical to PLP except for the removal of amino acids 116 through 150 [24]. DM-20 mRNA has been detected by RT-PCR in the murine neural tube and spinal cord during embryonic development, whereas the PLP transcript is the predominant postnatal isoform [25], [26]. *In situ* hybridization experiments at E10 using a DM-20 anti-sense probe detected signal in both the CNS (neural tube within the diencephalic basal plate) and the PNS (trigeminal and spinal ganglia, and sympathetic ganglion chain) [1], [25]. Other studies have confirmed this observation, demonstrating the presence of PLP/DM-20 RNA transcript as early as E9.5 [2], [27]. DM-20 is therefore clearly present in cells long before the appearance of defined mature or even precursor oligodendrocytes. Previous studies have shown DM-20 outside of the glial cell lineage, with expression detected in diverse cell types, including various neuronal cell lines such as G-26, B104, NG108-15, NG18-TG, Neuro2A, PC12, and P19 [27], [28], [29]. PLP/DM-20 transcripts and/or protein have also been found in cells of the thymus, spleen and testicles [30], [31].

Recently, various groups have identified specific cellular subtypes within the murine CNS expressing *Plp* gene products at early embryonic and postnatal stages. One such study tracked the fate of NG2^+^ oligodendrocyte progenitors co-labeling for *Plp* promoter driven EGFP [13]. They demonstrated the ability of the progenitors to not only differentiate into myelinating oligodendrocytes, but also into astrocytes and neurons. Another study compared PLP/DM-20 transcript levels at P5 and adult stages, between whole murine cortex and isolated NG2-positive cells. PLP mRNA was detected in isolated NG2-positive cells at both time points, whereas DM-20 mRNA was amplified from whole cortex only [32]. Similar to our own experiments, Delauney and colleagues crossed *Plp-Cre* mice to a GFP reporter, subsequently identifying GFP-positive neuroepithelial cells at E9.5, with the expressing profile switching to reporter-positive radial glial cells by E13.5 [3].

Thus, it is clear that the two *Plp* gene products yield varying and different expression profiles, and can be generally classified as follows – *Plp,* while expressed early in various CNS cellular subtypes, is at its highest levels postnatally and in an oligodendrocyte-specific manner, while *DM-20* is preferentially expressed early and diffusely in the embryo. It is therefore important to recognize the possibility for *Plp* promoter driven transgene expression simply as a by-product through activation of factors normally involved in DM-20 regulation.

Interestingly, in 1999 a novel exon was discovered lying within intron 1 of the *Plp* gene. Termed exon 1.1, it produced a protein with an additional 12 amino acid leader sequence [33]. The additional sequence allowed for expression of two further *Plp* variants, termed sr-PLP and sr-DM-20. These proteins have been detected in oligodendrocytes, muscle, and to an even larger extent in neurons [11], [14], [33]. Two similar novel exons have recently been discovered in intron 1 of the human *Plp1* gene, giving rise to unique PLP isoforms [34]. The isoforms are expressed predominantly in neurons and to lesser extent in oligodendrocytes, beginning during human fetal development. The authors suggest a role for the proteins in axonal-glial communication, the disruption of which would account for neuronal dysfunction in humans carrying *Plp1* mutations.

The very early and widespread expression of our transgene product forces us to reconsider certain aspects of *Plp* gene regulation. Perhaps there are regulatory elements activated differentially for each of the *Plp* derived isoforms, responsible for the specific spatial and temporal expression patterns observed during embryonic and postnatal development. Recent evidence suggests that this could indeed be the case. Within the *Plp* gene, six evolutionary conserved non-protein coding sequences were identified as *Plp/DM-20* enhancers, five of which were located within intron 1 [16]. When inserted as single copies driving expression of an *EGFPLacZ* reporter gene, each unique regulatory sequence matched characterized components of the *Plp* promoter's spatio-temporal expression profile as seen through a spectra of glial and neuronal lineage patterning.

The present study informs on *Plp* promoter functionality as a driver for both Cre and other transgene expression. Since the goal of many such studies is oligodendrocyte targeting within the CNS, it is important to better understand differences in gene regulation between *Plp* splice variants. Once more information is obtained deciphering the different regulatory elements governing expression of *Plp*/*DM-20* and sr-*Plp*/*sr-DM-20*, one could perhaps design a more specific promoter construct, which would allow for a postnatal, and more robust oligodendrocyte-specific expression of the requisite transgene. In the mean time, our work would stress that as long as one uses the tamoxifen inducible system (*Plp-CreER^t^*) and induces Cre activity at adult stages, one can achieve oligodendrocyte specific excision of target genes.

## Supporting Information

Figure S1 **Cre-recombinase is predominantly expressed in oligodendrocytes of P28** ***Plp-Cre*** **mouse optic nerves.** **A-I.** Optic nerve sections were double-stained with antibodies specific to CC-1 as a marker for mature oligodendrocytes (green), Cre (red) and counterstained with DAPI. Cre was not detected in WT optic nerve sections (B). Sections from transgenic mice of both F633 (D-F) and F627 (G-I) lines exhibited a similar pattern of Cre expression. High levels of Cre recombinase protein were detected in the CC1-positive oligodendrocytes. **J.** Optic nerve section stained for GFAP (green) as a marker for astrocytes, Cre (red) and counterstained with DAPI. A very small number of Cre-positive cells were also positive for GFAP. **K.** Optic nerve section stained for Ng2 (green) as a marker for OPCs, Cre (red) and counterstained with DAPI. A very small number of weakly stained Cre-positive cells co-labeled with Ng2 (arrow). Scale bar = 20 µm.
(TIF)

Figure S2 **Cre-recombinase is expressed both in and outside of the oligodendrocyte lineage in the spinal cord of** ***Plp-Cre*** **mice from line 633.** **A-I.** Ventral spinal cord sections from *Plp-Cre* line 633 mice were double-stained with antibodies specific to Cre (red) recombinase and either CC1 (green) for mature oligodendrocytes (A–C), Ng2 (green) for OPCs (D–F) or GFAP (green) for astrocytes (G–I) and counterstained with DAPI. Similar to line 627 many Cre-positive cells were CC-1-positive, however, a large percentage also co-stained for Ng2 and GFAP. Examples of Cre and glial marker co-labeling are denoted by arrows. WM  =  white matter, GM  =  grey matter. Scale bar = 20 µm.
(TIF)

Figure S3 **Cre-recombinase is not expressed in spinal cord neurons of P4** ***Plp-Cre*** **mice.** **A**–**I.** Ventral spinal cord sections were double-stained with antibodies specific to NeuN (green) as a marker for mature neurons, Cre (red), and counterstained with DAPI. Cre was not detected in WT sections (B). The low level puncti signal in the alpha motor neurons is background fluorescence. Sections from transgenic mice of both 633 (D–F) and 627 (G–I) lines exhibited a similar pattern of Cre expression. Cre-positive cells did not co-label with NeuN. Scale bar = 20 µm.
(TIF)

Figure S4 **Cre-recombinase is expressed both in and outside of the oligodendrocyte lineage in the cerebellum of** ***Plp-Cre*** **mice from line 633.** **A**–**F.** Deep cerebellar white matter regions from *Plp-Cre* line 633 mice were double-stained with antibodies specific to Cre (red) and either CC-1 (green) as a marker for mature oligodendrocytes (A–C), or Ng2 (green) as a marker for OPCs (D–F) and counterstained with DAPI. Similar to line 627, a large percentage of Cre-positive cells within the region co-stained for CC-1 and Ng2. **G**–**I.** Cerebellar sections of the developing granular layer from *Plp-Cre* line 633 mice were double-stained with antibodies specific to Cre (red), the neuronal marker NeuN (green) and counterstained with DAPI. Similar to line 627, many Cre-positive cells co-stained for NeuN. Examples of Cre-positive cells co-labeling with neuronal or glial markers are denoted by arrows. Scale bar = 20 µm.
(TIF)

Figure S5 **Cre-recombinase is expressed in cerebellar astrocytes of P4** ***Plp-Cre*** **mice.** **A**–**I.** Deep cerebellar white matter region sections were double-stained with antibodies specific to GFAP (green) as a marker for astrocytes, Cre (red), and counterstained with DAPI. Cre was not detected in WT sections (B). Sections from transgenic mice of both 633 (D–F) and 627 (G–I) lines exhibited a similar pattern of Cre expression. Only a small number of Cre-positive cells co-stained with GFAP. Examples of GFAP-positive cells co-labeling with Cre are denoted by arrows. Scale bar = 20 µm.
(TIF)
