# Supplementary figures and images for: The Proteolipid Protein Promoter Drives Expression outside of the Oligodendrocyte Lineage during Embryonic and Early Postnatal Development
Source: PLoS One. 2011 May 10;6(5):e19772. doi: 10.1371/journal.pone.0019772 (PMC3091881; doi:10.1371/journal.pone.0019772)

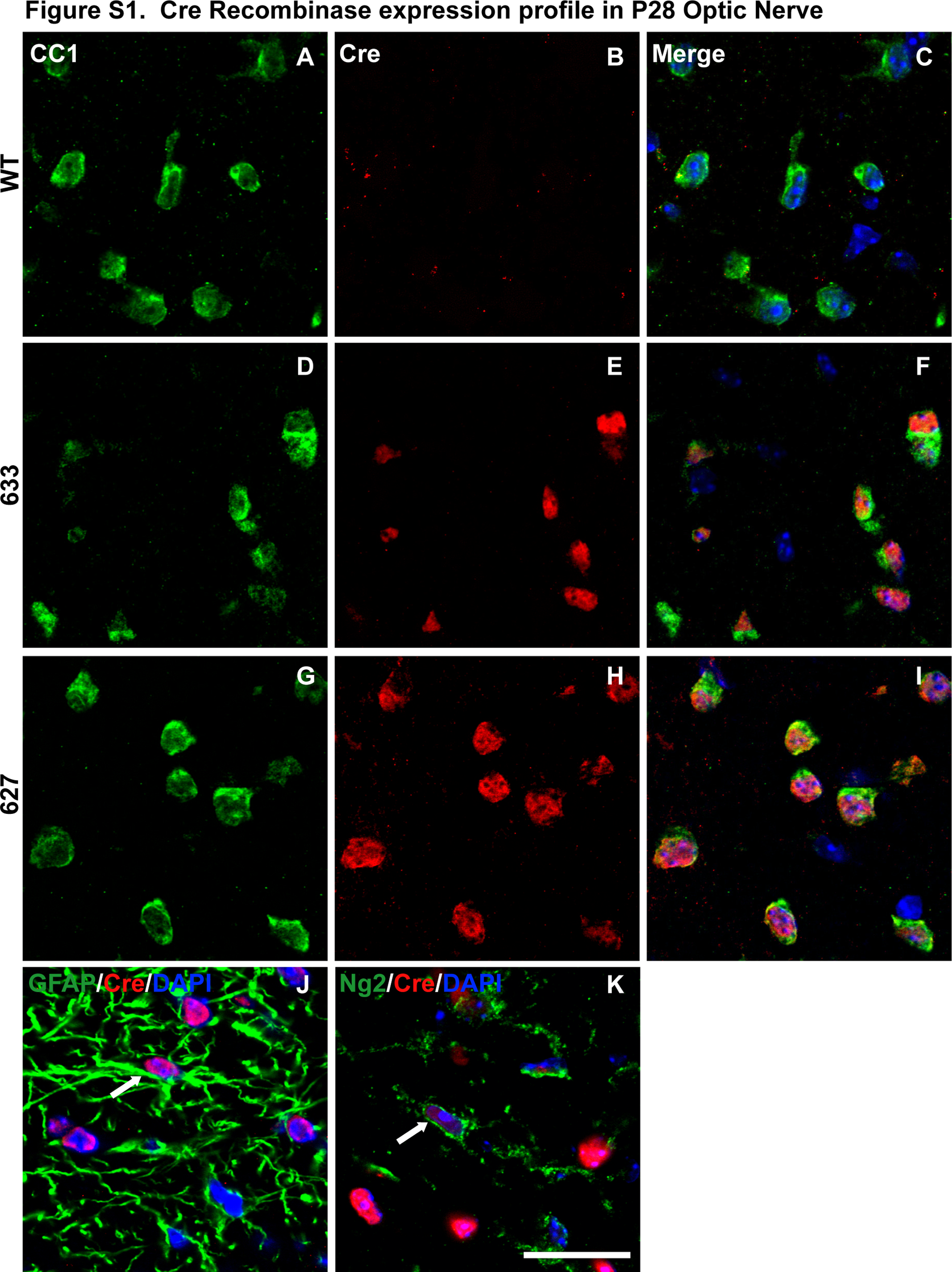

Supplement: Figure S1 — Cre-recombinase is predominantly expressed in oligodendrocytes of P28 Plp-Cre mouse optic nerves. A-I. Optic nerve sections were double-stained with antibodies specific to CC-1 as a marker for mature oligodendrocytes (green), Cre (red) and counterstained with DAPI. Cre was not detected in WT optic nerve sections (B). Sections from transgenic mice of both F633 (D-F) and F627 (G-I) lines exhibited a similar pattern of Cre expression. High levels of Cre recombinase protein were detected in the CC1-positive oligodendrocytes. J. Optic nerve section stained for GFAP (green) as a marker for astrocytes, Cre (red) and counterstained with DAPI. A very small number of Cre-positive cells were also positive for GFAP. K. Optic nerve section stained for Ng2 (green) as a marker for OPCs, Cre (red) and counterstained with DAPI. A very small number of weakly stained Cre-positive cells co-labeled with Ng2 (arrow). Scale bar = 20 µm. (TIF) [file pone.0019772.s001.tif]

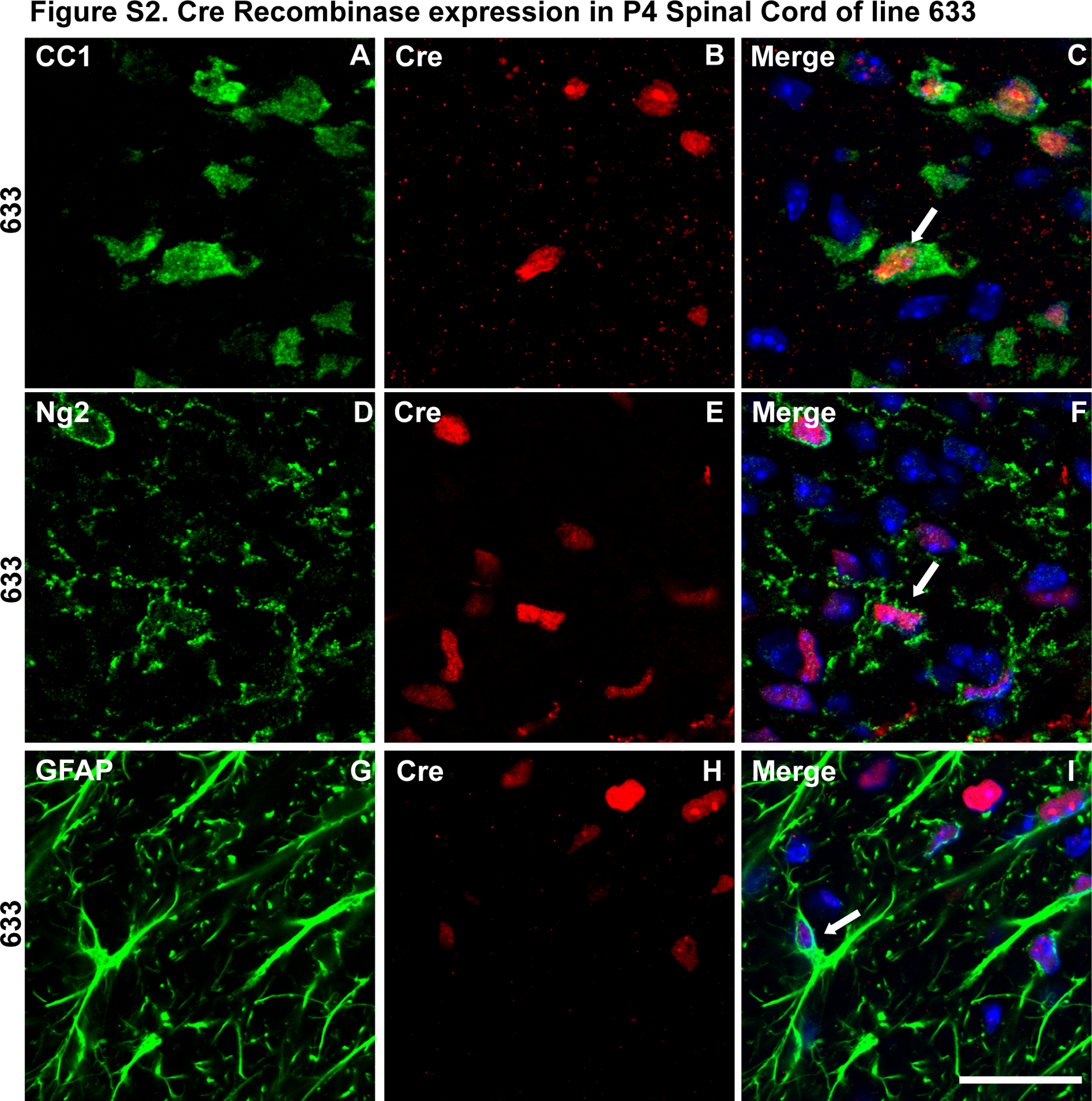

Supplement: Figure S2 — Cre-recombinase is expressed both in and outside of the oligodendrocyte lineage in the spinal cord of Plp-Cre mice from line 633. A-I. Ventral spinal cord sections from Plp-Cre line 633 mice were double-stained with antibodies specific to Cre (red) recombinase and either CC1 (green) for mature oligodendrocytes (A–C), Ng2 (green) for OPCs (D–F) or GFAP (green) for astrocytes (G–I) and counterstained with DAPI. Similar to line 627 many Cre-positive cells were CC-1-positive, however, a large percentage also co-stained for Ng2 and GFAP. Examples of Cre and glial marker co-labeling are denoted by arrows. WM = white matter, GM = grey matter. Scale bar = 20 µm. (TIF) [file pone.0019772.s002.tif]

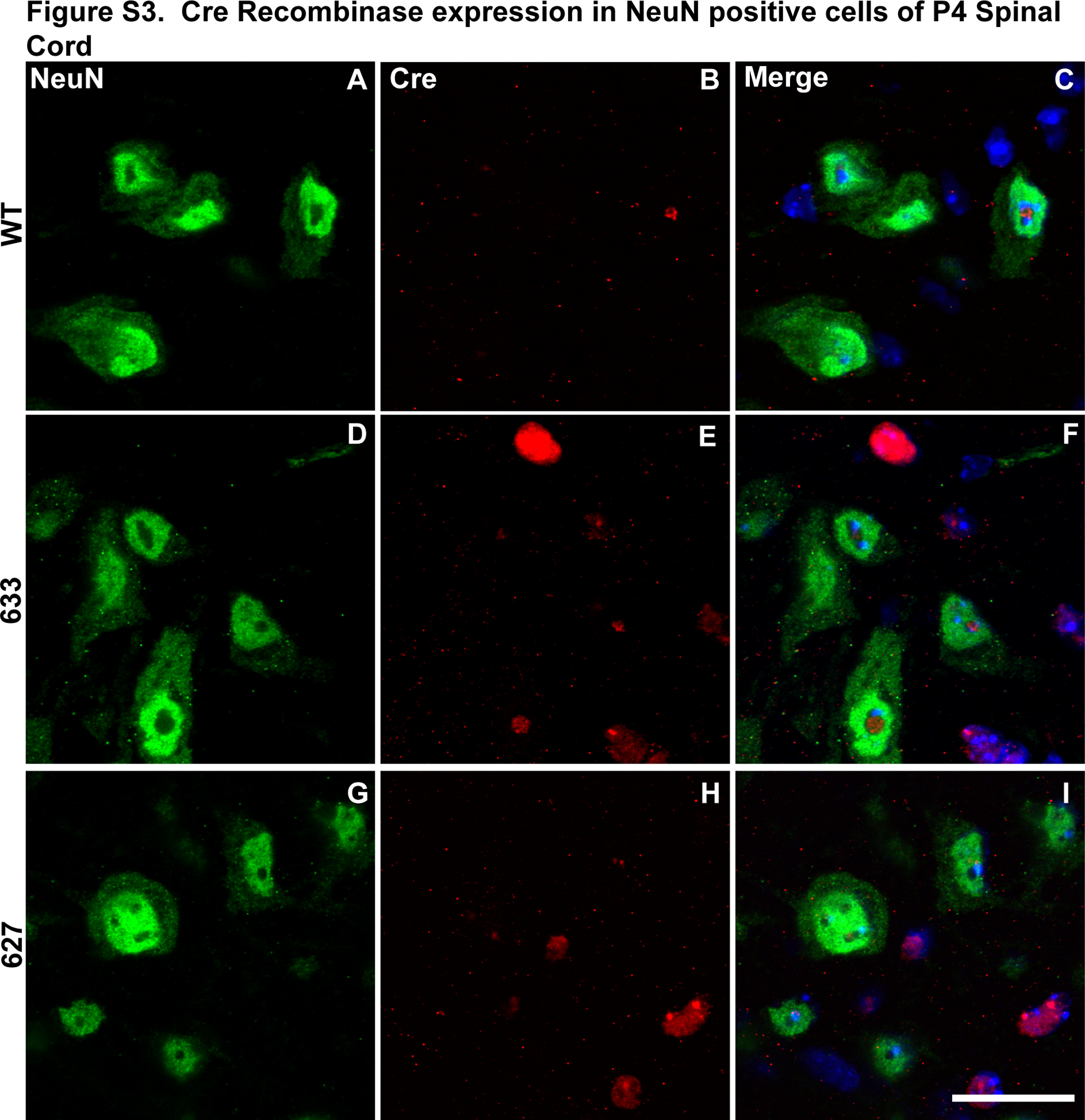

Supplement: Figure S3 — Cre-recombinase is not expressed in spinal cord neurons of P4 Plp-Cre mice. A–I. Ventral spinal cord sections were double-stained with antibodies specific to NeuN (green) as a marker for mature neurons, Cre (red), and counterstained with DAPI. Cre was not detected in WT sections (B). The low level puncti signal in the alpha motor neurons is background fluorescence. Sections from transgenic mice of both 633 (D–F) and 627 (G–I) lines exhibited a similar pattern of Cre expression. Cre-positive cells did not co-label with NeuN. Scale bar = 20 µm. (TIF) [file pone.0019772.s003.tif]

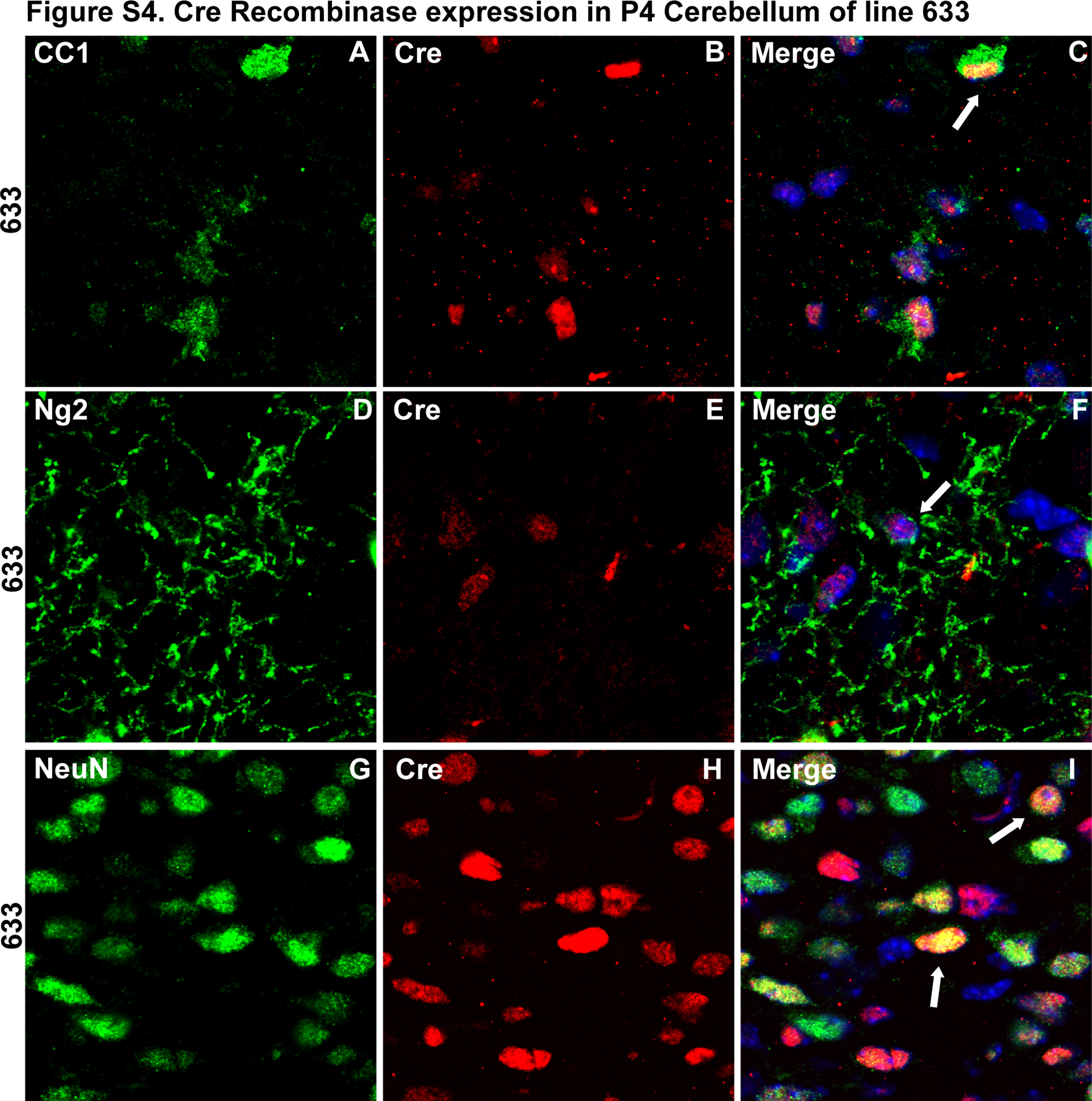

Supplement: Figure S4 — Cre-recombinase is expressed both in and outside of the oligodendrocyte lineage in the cerebellum of Plp-Cre mice from line 633. A–F. Deep cerebellar white matter regions from Plp-Cre line 633 mice were double-stained with antibodies specific to Cre (red) and either CC-1 (green) as a marker for mature oligodendrocytes (A–C), or Ng2 (green) as a marker for OPCs (D–F) and counterstained with DAPI. Similar to line 627, a large percentage of Cre-positive cells within the region co-stained for CC-1 and Ng2. G–I. Cerebellar sections of the developing granular layer from Plp-Cre line 633 mice were double-stained with antibodies specific to Cre (red), the neuronal marker NeuN (green) and counterstained with DAPI. Similar to line 627, many Cre-positive cells co-stained for NeuN. Examples of Cre-positive cells co-labeling with neuronal or glial markers are denoted by arrows. Scale bar = 20 µm. (TIF) [file pone.0019772.s004.tif]

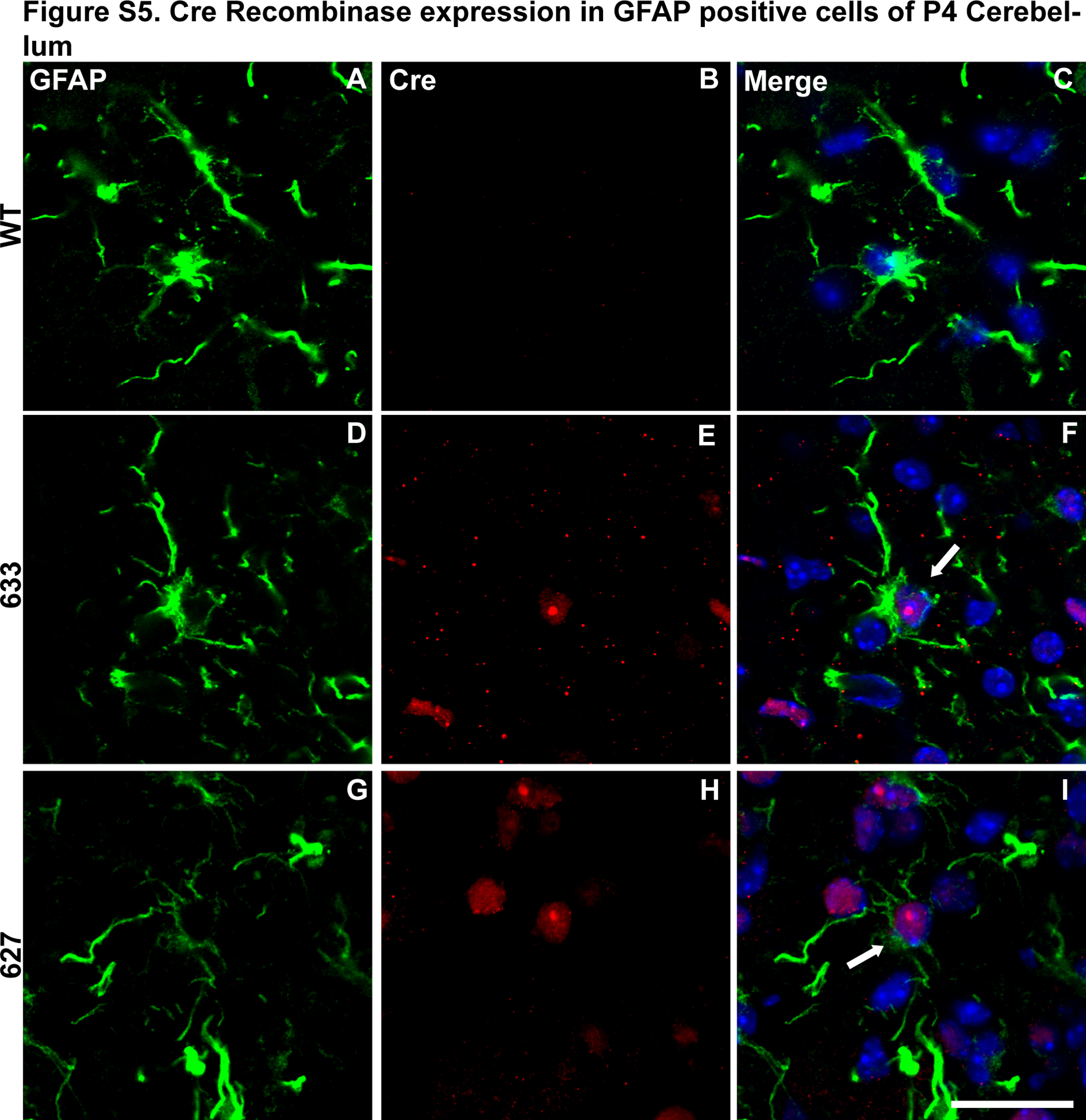

Supplement: Figure S5 — Cre-recombinase is expressed in cerebellar astrocytes of P4 Plp-Cre mice. A–I. Deep cerebellar white matter region sections were double-stained with antibodies specific to GFAP (green) as a marker for astrocytes, Cre (red), and counterstained with DAPI. Cre was not detected in WT sections (B). Sections from transgenic mice of both 633 (D–F) and 627 (G–I) lines exhibited a similar pattern of Cre expression. Only a small number of Cre-positive cells co-stained with GFAP. Examples of GFAP-positive cells co-labeling with Cre are denoted by arrows. Scale bar = 20 µm. (TIF) [file pone.0019772.s005.tif]
